# The Association of Plasma-Free Branched-Chain Amino Acids with Disease Related Parameters in Ulcerative Colitis

**DOI:** 10.3390/diagnostics10100798

**Published:** 2020-10-08

**Authors:** Efstathia Papada, Charalampia Amerikanou, Aristea Gioxari, Nick Kalogeropoulos, Andriana C. Kaliora

**Affiliations:** Department of Dietetics and Nutritional Science, School of Health Science and Education, Harokopio University, 70 El. Venizelou Ave, 17671 Athens, Greece; efpapada@gmail.com (E.P.); amerikanou@windowslive.com (C.A.); arisgiox@gmail.com (A.G.); nickal@hua.gr (N.K.)

**Keywords:** BCAAs, ulcerative colitis, inflammation, calprotectin

## Abstract

Branched-chain amino acids (BCAAs) are involved in immune system’s metabolic pathways and play fundamental role in gut health. Our aim was to assess BCAA plasma levels in patients with ulcerative colitis (UC) and associations of plasma BCAAs with disease-related parameters. This was a case-control study in adult patients with UC and BMI-matched controls. A total of 150 volunteers were screened between May 2016 and June 2017; 43 patients and 34 healthy controls were enrolled. Medical and dietary history (3 × 24 h recalls, MedDiet score), anthropometric measurements, blood and fecal samples were collected. We measured BCAAs in plasma with gas chromatography-mass spectrometry. In patients, fecal calprotectin, lactoferrin, lysozyme and defensin were quantified. Dietary pattern was similar in patients and controls. Plasma-free BCAA profiles did not differ between groups. Regression analysis showed that i) valine was inversely associated with calprotectin (*p* = 0.007) and ii) isoleucine with age (*p* = 0.031), after adjusting for age, sex, PMS and smoking. Leucine was negatively associated with age (*p* = 0.015) after adjusting for age, sex and PMS, but this association vanished when smoking was introduced. No correlation was observed between total BCAAs with any of the parameters. Plasma-free valine is negatively associated with calprotectin in patients with UC.

## 1. Introduction

Ulcerative colitis (UC) is a chronic inflammatory gastrointestinal ailment characterized by periods of relapse and remission, while patients experience a compromised health-related quality of life, economic productivity and social function [1].

Apart from successful medical management with a variety of pharmacological agents, diet seems to play a crucial role in the progression and management of UC [2]. Not only dietary regimens have been brought under investigation, such as FODMAPs, Mediterranean diet and the Specific Carbohydrates Diet (SCD), but also the role of specific micronutrients [3,4]. However, the research gaps about the role of dietary elements and regimens in the management of the disease are still present. 

Amino acids act as regulators in a variety of metabolic pathways, controlling and maintaining intestinal health. They are considered as structural elements of protein synthesis and participate in various mechanisms such as cellular communication, gene expression, oxidative stress, immune process and intracellular protein metabolism [5]. In some studies, plasma amino acid profile in patients with ulcerative colitis differs from healthy individuals, suggesting a potential association with disease progression, diagnosis and treatment [6].

The immune system depends highly on the synthesis of proteins for the production of cells, immunoglobulins, cytokines and their receptors; Branched-Chain Amino Acids (BCAAs), namely leucine, isoleucine, valine, are necessary for the abovementioned processes. It is therefore evident that inadequate levels of BCAAs may act as inhibitory factors and interfere with the normal immune response [7]. Herein, we evaluated the metabolic profile of plasma-free BCAAs in patients with UC and in healthy controls and we investigated their potential association with clinical parameters and fecal inflammatory biomarkers.

## 2. Materials and Methods

### 2.1. Ethical Considerations 

Harokopio University Ethics Committee (49/29-10-2015) approved the study protocol on 29 October 2015 and all participants provided a written Informed Consent. The study was conducted following the rules of the Declaration of Helsinki of 1975, revised in 2013. Standardized laboratory techniques were applied and the personnel worked according to Good Clinical Practice.

### 2.2. Participants 

In this case-control study, patients with ulcerative colitis (UC) and healthy controls (HC) were recruited between May 2016 and June 2017. Adult male and female outpatients with endoscopic diagnosis of UC were enrolled through an announcement to the Hellenic Society of Crohn’s Disease and Ulcerative Colitis patients. The Partial Mayo Score (PMS) was used to assess disease activity [8]. UC patients were included in the study. Exclusion criteria were suffering from other inflammatory bowel diseases, e.g., Crohn’s disease, positive stool culture for enteric pathogens or Clostridium difficile toxin, bowel surgery ≤3 months prior to screening, colostomy, enteral or parenteral nutrition, alcohol or drug abuse, food supplement use, a vegan or macrobiotic diet in the 5 years prior to screening, any malignancy in the year prior to screening or cancer survivors <10 years, the presence of other chronic diseases, serious cardiovascular disease, diabetes, peptic ulcer, pregnancy, and lactation. HC were selected to match for BMI with UC patients. The study took place in Athens, Greece.

### 2.3. Assessment of Patients 

#### 2.3.1. Medical History

Medical history was recorded by a gastroenterologist and included general information, as well as disease specific data (i.e., brief history of UC, age of diagnosis, complications, and treatment). Disease severity was evaluated using the PMS. 

#### 2.3.2. Dietary Assessment

A total of three 24-h recall dietary intakes (2 weekdays, 1 weekend day) were recorder by an experienced dietitian for patients with UC and for healthy controls. The software package Nutritionist Pro™ (Axxya Systems, Stafford, Texas 77477, USA) was used to analyse dietary intakes. Patients with UC and HC were under no special diet. Overall diet quality was assessed based on adherence to the Mediterranean Diet (MD) using the MedDiet scoring method, which is based on the inherent characteristics of the MD. According to this rating scale, the MedDiet score ranges from 0 to 55 with higher values indicating greater adherence [9].

#### 2.3.3. Quality of Life Assessment

Quality of life was evaluated with the Inflammatory Bowel Disease Questionnaire (IBDQ). The IBDQ includes 32 questions about bowel habit, social, systemic and emotional performance and is scored from 32 to 224 points. Higher scores indicate a better quality of life [10].

#### 2.3.4. Blood Sample Collection 

Standard blood sampling (20mL) was undertaken by the study personnel. After collection, blood samples were centrifuged at 3000 rpm for 10 min at 4 °C for plasma and serum isolation. All samples were stored at −80 °C until further analysis. 

#### 2.3.5. Anthropometric Assessment

Body weight was measured to the nearest 0.1 kg twice. Height was measured with a standard stadiometer to the nearest millimeter twice. Body Mass Index (BMI) was also calculated. 

### 2.4. Laboratory Analyses 

#### 2.4.1. Biochemical Analyses

Serum albumin was quantified as a measure of nutritional status and C-reactive protein (CRP) as a measure of inflammation using a biochemical analyzer.

#### 2.4.2. Stool Inflammatory Biomarkers

A stool preparation system filled with extraction buffer IDK Extract^®^ (Immundiagnostik, AG, Bensheim, Germany) was provided to the participants for stool sample collection. Stool extracts were kept for a maximum of 9 days at −20 °C until further analysis. Levels of calprotectin, lysozyme, lactoferrin and defensin were evaluated in stool samples (Immundiagnostik, AG, Bensheim, Germany).

#### 2.4.3. Amino Acids Profile

We applied Gas Chromatography–Mass Spectrometry (GC-MS) to profile the AA in plasma samples in an Agilent (Wallborn, Germany) series GC 6890 N gas chromatograph, coupled with an HP 5973 Mass Spectrometer detector (EI, 70 eV), split-splitless injector and an HP 7683 autosampler. The extraction and derivatization of AAs in plasma was conducted as described in the Phenomenex EZ:Faast™ Free (Physiological) Amino Acid Analysis by GC-MS manual (Phenomenex UK, Ltd., Macclesfield, UK). An aliquot (2 μL) of the derivatized samples was injected into the GC at a split ratio of 1:15. AAs separation was accomplished using a Phenomenex Zebron ZB-A AA analysis dedicated column (length = 10 m, internal diameter = 0.25 mm, film thickness = 25 μm). Carrier gas was high purity helium at constant flow of 1.1 mL/min. The injector and transfer line temperatures were 250 and 340 °C, respectively. Initial oven temperature was 110 °C, increased to 320 °C at 30 °C/min and held at 320 °C for 3 min. We applied a selective ion monitoring (SIM) GC/MS method for the detection of 26 AAs, based on the ±0.05 retention time (Rt) presence of a target and a qualifier ion, with the exception of β-aminoisobutyric acid with more than one qualifier ions. Quantification was carried out using norvaline as internal standard and creating reference curves for every AA by means of standard solutions.

Additionally, to plasma levels of valine, leucine and isoleucine, total BCAAs were also calculated by summing leucine, isoleucine and valine levels.

### 2.5. Statistical Analysis

Data are expressed as mean ± standard deviation (SD) and the qualitative data as absolute values. Kolmogorov–Smirnov test was used to assess normal distribution. The differences between two independent groups were analyzed using Student’s t-test for normally distributed variables and Mann–Whitney U test for those not normally distributed. Spearman’s correlation tests were used for the correlation analysis. When bivariate correlations were significant, we also performed multivariate linear regression models, with BCAA levels being log transformed. Level of significance was set at 0.05. Statistical analysis was conducted with the SPSS software (SPSS for Windows, version 21.0, SPSS Inc., Chicago, IL, USA).

## 3. Results

A total of 43 patients (39.0 ± 12.0 years old) diagnosed with UC met our inclusion criteria and were enrolled in the study. In the group of HC, 34 participants (26.2 ± 5.4 years old) were included. Table 1 shows the demographics, disease characteristics, biochemical and fecal biomarkers of the participants. In UC group, the mean PMS was 2.4 (± 2.1) indicating moderate disease activity [8]. The mean IBDQ score assessing quality of life was below the threshold of 168 points that indicates relapse [10]. The majority of patients were on treatment with mesalazine, whereas only fourpatients received no treatment. Mean albumin and CRP values were within the normal range. Regarding fecal biomarkers, there is still controversy about the optimal cut-off points determining remission. However, as shown on the table, the mean values for calprotectin and lactoferrin were above the suggested thresholds of remission [11].

The analysis of dietary intakes and MedDiet Score of UC patients and HC is given in Table S1. There were no significant differences between the groups. The mean dietary intake of protein, saturated fats and fibers that may affect plasma BCAAs [12], were similar to patients and control. No difference in MedDiet score was reported either, indicative of an alike diet quality.

Table 2 presents the plasma levels of BCAAs valine, leucine and isoleucine, as well as the total BCAAs in patients with UC and in healthy controls. No significant differences were observed between the two groups neither in individual BCAAs nor in the total BCAAs content of plasma.

Correlation analyses were conducted for plasma BCAAs with disease related parameters and the associations are presented in Table 3. Valine levels were negatively correlated with calprotectin and positively with BMI. Leucine was negatively correlated with age and positively with BMI and isoleucine negatively with age. No correlation was observed between the total BCAAs content with any of the parameters.

The associations between BCAAs and parameters that exhibited significant correlations are shown in Table 4: (a) calprotectin was inversely associated with valine (*p* = 0.007) and (b) age with isoleucine (*p* = 0.031), after adjusting for age, sex, PMS and smoking status (model 4). BMI was not associated with neither valine (beta = 0.001, *p* = 0.817) nor leucine (beta = 0.000, *p* = 0.936). Finally, leucine was negatively associated with age when adjusting for age, sex and PMS (model 3), but the significance disappeared when adding smoking status in the model.

## 4. Discussion

UC affects people of all life stages with its incidence and prevalence increasing dramatically worldwide. A combination of genetic, environmental and immunological factors seems to contribute to the onset of the disease and several molecular mechanisms have been proposed to understand the pathogenesis and progress of the disease. Suppression of mitochondrial genes and function [13], disruption of the intestinal mucosal barrier and bacterial invasion resulting in intestinal inflammation, and further TLR4/NF-κB stimulation in intestinal epithelial cells are some of the proposed mechanisms [14]. The role of intestinal microbiota has also been investigated and there seems to be evidence on differences in the intestinal microbial metabolism between healthy and patients with IBD [15] as well as on the involvement of bacterial sulfate metabolism in gut inflammation [16,17,18] More specifically, sulphate reducing bacteria use sulfate as an electron acceptor in dissimilatory sulfate reduction and the final product is H2S, a potential toxic, mutagenic and cancerogenic compound [19].

Amino acids have attracted the research interest as they possibly play a role in the development and diagnosis of IBD. They are major components for protein synthesis and participate in various pathways of inflammation, immune response and metabolism. Herein, we evaluated the plasma-free BCAAs, valine, leucine, isoleucine, and their association with clinical and stool inflammatory markers in UC patients. It is worth mentioning that numerous studies have shown the association of BCAAs with other chronic inflammatory conditions such as diabetes and insulin resistance, obesity and cardiovascular disease [20].

When investigating an association of BCAAs with fecal calprotectin, lysozyme, lactoferrin or defensin, we detected a negative association of plasma valine with fecal calprotectin after adjusting for age, sex, disease activity and smoking status. Fecal biomarkers are strongly correlated with inflammation in IBD as they are produced closer to the location of inflammation. Fecal calprotectin, lysozyme, lactoferrin and defensin are the most frequently used biomarkers in research, with fecal calprotectin being also used in clinical practice. Calprotectin is a non-invasive biomarker, more specific of mucosal inflammation than CRP or erythrocyte sedimentation rate, which is less influenced by other non-intestinal conditions. It is widely used in clinical practice such as in diagnosis of IBD, disease activity and therapy response assessment, as well as relapse prediction [16]. Previous research suggested that fecal calprotectin correlates with disease activity, especially in colonic IBD. To our knowledge, this is the first study showing that plasma valine is negatively associated with fecal calprotectin. This is an intriguing result, since previous research has shown that another plasma amino acid, namely citrulline, did not correlate with fecal calprotectin or with CRP in pediatric and adolescent IBD, known as direct markers of inflammation [21]. Herein, the negative association of valine with fecal calprotectin was not accompanied by an association of total or individual BCAAs with IBDQ or with PMS or CRP that are widely used in clinical practice in order to assess and monitor disease activity. Previous studies in other inflammatory conditions such as metabolic syndrome [22], cardiovascular disease [23], type 2 diabetes [24] and chronic kidney disease [25] pointed towards an association of BCAAs with inflammation and disease severity. However, the findings of Van Waardenburg and colleagues did not show any association of BCAAs with CRP in critical illness coming into agreement with our findings [26].

When we assessed the association of BCAAs with patients’ characteristics, isoleucine and leucine, were negatively associated with age. In our study, enrolled patients and HC were in young adulthood to middle ages and no old aged were included. Isoleucine and leucine may play a distinct role for supporting skeletal muscle activity and a previous metabolomics study showed that isoleucine and leucine levels decrease with aging [27]. In the work of Flores-Guerrero and coworkers in 6244 subjects significantly elevated plasma BCAAs were reported in old-aged participants and the association with metabolic disorders was independent of age [28].

Our protocol has both strengths and limitations. According to our knowledge this is the first study that has evaluated plasma levels of BCAAs in patients with UC and their relationship with patients’ stool inflammatory biomarkers. The use of validated tools to assess the selected parameters is an additional strength, such as the IBDQ, which is the most widely used tool to assess the quality of life in IBD patients [29]. Additionally, no older-aged participants were included to avoid confounding the real relationship between BCAAs and UC characteristics. Limitations include its observational design that does not allow determining the causality of the associations. Additionally, herein plasma-free BCCAs were evaluated instead of whole blood levels that represent both plasma and intracellular amino acids in blood cells.

## 5. Conclusions

BCAAs seem to play a fundamental role in controlling and maintaining intestinal health. In UC particularly, valine is associated with inflammation localized to the bowel. Although the mechanisms underlying the association of BCAAs to disease severity need to be elucidated, our study supports the significance of BCAAs as emerging biomarkers in UC.

## Figures and Tables

**Table 1:** Characteristics of patients with UC and HC.

	UC (*n* = 43)	HC (*n* = 34)
**BMI (kg/m2)**	24.5 ± 5.2	24.4 ± 4.1
≤29.9	38	30
≥30.0	5	4
**Marital status**		
Single	17	29
Married	23	5
Divorced	3	
**Disease activity**		
PMS	2.4 ± 2.1	-
**IBDQ**	160.9 ± 35.4	
**Disease duration (yrs)**	8.9 ± 6.9	-
**Treatment**		
Mesalazine	35	-
Azathioprine	10	-
Corticosteroids	10	-
No treatment	4	-
**Albumin (g/dL)**	4.0 ± 1.1	5.0 ± 0.4
**CRP (mg/dL)**	3.9 ± 5.4	-
**Calprotectin (μg/g)**	1921.4 ± 4284.4	-
**Lactoferrin (μg/g)**	154.6 ± 216.5	-
**Lysozyme (μg/g)**	19.8 ± 27.3	-
**Defensin (ng/g)**	32.6 ± 66.4	-

Values are presented as mean ± SD. UC, ulcerative colitis; HC, healthy control; PMS, partial mayo score; IBDQ, inflammatory bowel disease questionnaire; CRP, C-reactive protein.

**Table 2:** Comparison of plasma branched-chain amino acids (BCAAs) between the UC patients and HC.

	UC (*n* = 43) Mean ± SD	HC (*n* = 34) Mean ± SD	*p*
**Valine (nmol/L)**	333.7 ± 85.1	348.6 ± 86.4	0.450
**Isoleucine (nmol/L)**	60.5 ± 22.5	62.1 ± 19.9	0.237
**Leucine (nmol/L)**	121.2 ± 34.2	130.2 ± 30.5	0.752
***Total BCAAs (nmol/L)***	515.4 ± 136.0	540.9 ± 131.1	0.467

Values are presented as mean ± SD. UC, ulcerative colitis; HC, heathy controls; BCAAs, branched-chain amino acids. Differences between groups were analyzed by Mann–Whitney U test or Student’s test. Difference was considered significant at *p* < 0.05.

**Table 3:** Correlation of plasma BCAAs with clinical indices, stool inflammatory biomarkers and age of patients with UC.

	Valine	Leucine	Isoleucine	Total BCAAs				
	*Correlation coefficient*	*p*	*Correlation coefficient*	*p*	*Correlation coefficient*	*p*	*Correlation coefficient*	*p*
**Age (years)**	−0.169	0.063	−0.190	**0.036**	−0.184	**0.043**	−0.233	0.168
**ΒΜΙ (kg/m^2^)**	0.201	**0.027**	0.185	**0.043**	0.124	0.178	0.046	0.779
**IBDQ**	0.052	0.574	0.02	0.833	−0.039	0.677	−0.044	0.786
**PMS**	0.106	0.508	0.165	0.304	0.226	0.155	0.127	0.441
**Calprotectin (μg/g)**	−0.388	**0.016**	−0.180	0.28	−0.165	0.33	−0.307	0.064
**Lactoferrin (μg/g)**	−0.145	0.422	0.02	0.91	0.093	0.611	−0.073	0.69
**Lysozyme (μg/g)**	−0.286	0.087	−0.210	0.213	−0.190	0.266	−0.252	0.139
**Defensin (ng/g)**	−0.170	0.438	−0.014	0.948	−0.090	0.689	−0.142	0.527

UC, ulcerative colitis; BCAAs, branched-chain amino acids; IBDQ, inflammatory bowel disease questionnaire; PMS, Partial Mayo score. Spearman’s correlation tests were used for the correlation analysis. Statistically significant level was set at *p < 0.05*. Values in bold indicate statistical significance

**Table 4:** Multivariate regression analysis addressing the associations between BCAAs with patient characteristics.

Ulcerative Colitis								
	Model 1		Model 2		Model 3		Model 4	
Valine *								
	**B**	*p*	**B**	*p*	**B**	*p*	**B**	*p*
**Calprotectin (μg/g)**	−1967*10^−5^	0.094	−1759*10^−5^	0.115	−2419*10^−5^	**0.026**	−2796*10^−5^	**0.007**
Leucine *								
	**B**	*p*	**B**	*p*	**B**	*p*	**B**	*p*
**Age (years)**	−0.003	0.072	−0.003	**0.044**	−0.004	**0.015**	−0.003	0.057
Isoleucine *								
	**B**	*p*	**B**	*p*	**B**	*p*	**B**	*p*
**Age (years)**	−0.005	**0.023**	−0.005	**0.020**	−0.006	**0.006**	−0.004	**0.031**

BCAAs, branched-chain amino acids; Model 1: Unadjusted model; Model 2: Adjustment for age. Sex; Model 3: Adjustment for age, sex, Partial Mayo Score; Model 4: Adjustment for age, sex, Partial Mayo Score, smoking status; * log transformed; Level of significance was set at *p* < 0.05. Values in bold indicate statistical significance.

## Supplementary Materials

The following are available online at https://www.mdpi.com/2075-4418/10/10/798/s1, Table S1: Dietary intakes and MedDiet score of patients with UC and of HC.
