# Supplementary material for: The Association of Plasma-Free Branched-Chain Amino Acids with Disease Related Parameters in Ulcerative Colitis
Source: Diagnostics (Basel). 2020 Oct 8;10(10):798. doi: 10.3390/diagnostics10100798 (PMC7600496; doi:10.3390/diagnostics10100798)
Supplement: Supplementary file 1 [file diagnostics-10-00798-s001.pdf]

Table S1. Dietary intakes and MedDiet score of patients with UC and of HC.

	**UC patients**<br>**Mean ± SD**	**HC**<br>**Mean ± SD**	**P**
**Energy (kcal)**	1868.5±760.4	2164.3±1279.1	0.488
**Protein (g)**	91.0 ±48.7	81.5±34.7	0.652
**Carbohydrates (g)**	223.0 ±135.8	238.5±98.5	0.313
**Fats (g)**	76.0± 42.6	96.7±113.8	0.971
**Saturated FA (g)**	22.8±13.3	27.0±2.6	0.354
**Monounsaturated FA (g)**	33.4±21.4	43.0±81.2	0.603
**Polyunsaturated FA (g)**	12.7±9.9	13.3±14.7	0.846
**Fiber (g)**	12.3±11.4	18.9±14.2	0.051
**MedDiet score**	30.0±5.7	32.3±5.8	0.133

Values are presented as mean±SD. Differences between groups were analysed by Mann-Whitney U test or Student's test. Difference was considered significant at $P<0.05$.
